# Microglia in Alzheimer Brain: A Neuropathological Perspective

**DOI:** 10.1155/2012/165021

**Published:** 2012-05-13

**Authors:** Robert E. Mrak

**Affiliations:** Department of Pathology, University of Toledo College of Medicine, 3000 Arlington Avenue, Toledo, OH 43614, USA

## Abstract

Microglia have long been noted to be present and activated in Alzheimer brain. Demonstrations that these microglia are associated with the specific lesions of Alzheimer disease—A*β* plaques and neurofibrillary tangles—and that these microglia overexpress the potent proinflammatory cytokine interleukin-1 led to the recognition of a potential pathogenic role for these cells in initiation and progression of disease. Activated, cytokine-overexpressing microglia are near-universal components of A*β* plaques at early (diffuse) and mid (neuritic) stages of progression in Alzheimer brain, and only decline in end-stage, dense core plaques. They correlate with plaque distribution across cerebral cortical cytoarchitectonic layers and across brain regions. They also show close associations with tangle-bearing neurons in Alzheimer brain. Microglial activation is a consistent feature in conditions that confer increased risk for Alzheimer disease or that are associated with accelerated appearance of Alzheimer-type neuropathological changes. These include normal ageing, head injury, diabetes, heart disease, and chronic intractable epilepsy. The neuropathological demonstration of microglial activation in Alzheimer brain and in Alzheimer-related conditions opened the field of basic and applied investigations centered on the idea of a pathogenically important neuroinflammatory process in Alzheimer disease.

## 1. Introduction

Microglia have been known to be present in the characteristic plaques of Alzheimer disease since the first descriptions of these cells by del Rio Hortega and Penfield in the 1920s [1], but half a century would pass before attention returned to these cells. The first suggestion of a causative role for microglia in Alzheimer disease came from Glenner, who hypothesized in 1979 that the amyloid found in Alzheimer brain was produced by these cells [2]. This idea dominated several subsequent studies that identified microglia associated with amyloid plaques in the brains of Alzheimer patients [3–5]. The idea was largely abandoned when the neuronal origin of A*β* was elucidated [6], although occasional studies have returned to this idea [7].

**Figure 1:**
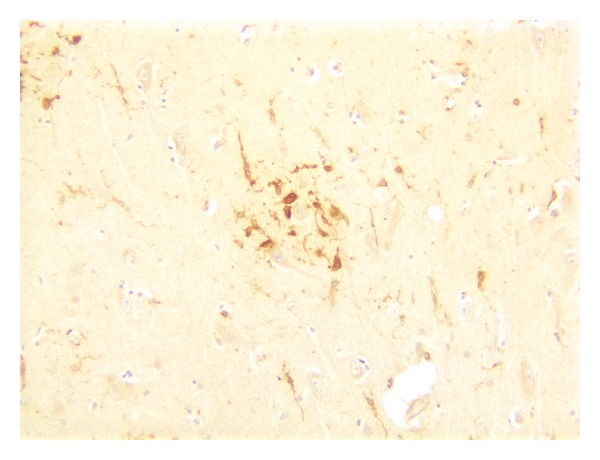
Activated microglia, overexpressing interleukin-1, within an A*β* plaque in Alzheimer brain. Immunohistochemistry using an antibody specific for IL-1*α*.

The first evidence that microglia may have an immunological—rather than a phagocytic or A*β*-processing—function in Alzheimer brain was the demonstration in 1989 by Griffin and colleagues that these microglia express the potent immunomodulatory cytokine interleukin-1 [8] (Figure 1). This report, together with the finding that interleukin-1 regulates the synthesis of the A*β* precursor protein [9], immediately suggested that microglia and their cytokines might play a role in driving plaque development, a concept very different from ideas about amyloid production or phagocytosis and protein degradation that had been previously attributed to microglia. Over the next several years, additional cytokines were added to the listing of proteins that are elevated in Alzheimer brain. These include interleukin-6 [10], transforming growth factor *β*_1_ [11], interferon *α* [12, 13], and interleukins-2 and -3 [14].

Ideas regarding the role of microglia in Alzheimer disease have continued to evolve over the 20 years since these seminal studies. Neuropathological investigations, in particular, have both suggested and supported ideas about the potential roles of inflammatory mechanisms in A*β* plaque formation and progression in Alzheimer disease, and the potential roles of microglial activation in progressive plaque-associated neuritic damage, neuronal damage, and neuronal death. This review will highlight these neuropathological studies.

## 2. Microglial Identification in Human Brain

Microglia were first described in 1899 by Nissl, who distinguished these cells from other neural components based on the shape of their nuclei [15]. The definitive identification and characterization of these cells were done in the 1920s by del Rio Hortega and Penfield, using a silver carbonate staining technique [1]. Microglia are now known to express a wide variety of immune-related molecules and antigens [16], many of which can be used to immunolabel microglia in histological tissue sections. “Resting” microglia, found throughout normal brain parenchyma, express many of these molecules either at very low levels or not at all.

In contrast to the low levels of expression of immune-related molecules by resting microglia, immunological challenge or tissue injury leads to upregulation of many of these factors, a process known as microglial activation. With further activation, microglia undergo morphological changes that include enlargement and withdrawal of their ramified processes. Activated microglia can be identified through their expression of such factors. In general, however, antibodies against secreted products such as interleukin-1*β* (IL-1*β*) or tumor necrosis factor-*α* generally yield poor results in paraffin sections as these soluble peptides are lost during tissue processing. In contrast, the cytokine IL-1*α* is expressed by microglia as a membrane-bound peptide, and immunohistochemistry using antibodies against IL-1*α* is very effective at labeling activated microglia while producing little or no labeling of resting microglia (Figure 1) [17]. Other techniques that have been used to identify microglia include MHC class II cell surface receptors [18], Fc receptors [19], various lectins [20–22], and other monocyte markers [23, 24]. More recently, immunohistochemistry for ionized calcium binding adapter molecule 1 (Iba1) has been identified as a reliable marker for microglia, although this technique labels resting as well as activated microglia and is thus not specific for activated forms [25, 26]. A subset of microglia express ferritin and can be immunolabelled with anti-ferritin antibodies. Such expression, however, appears to represent a degenerative, or “dystrophic” change in microglia rather than an activated state [27].

## 3. Microglial Associations with A***β*** Plaques

Activated microglia are near-universal components of A*β* plaques in Alzheimer brain. In Alzheimer brain, microglia accumulate fragmented DNA, presumably originating from neuronal injury and death [28]. Such accumulation, together with cytokine stimulation, is a potent microglial activating stimulus [28]. Microglial activation has been shown to progress with clinical (CDR) stage of dementia [29, 30], with neuropathological (Braak and Braak [31]) stage of disease severity [29], and with stage of progression of individual A*β* plaques [32]. The distribution of activated microglia across different brain regions closely parallels that of neuritic plaques in Alzheimer brain, with involvement of hippocampus > temporal lobe > frontal and occipital lobes [33]. Moreover, there is variation in the distribution of microglia in different cortical cytoarchitectonic layers [34]. In Alzheimer brain, this distribution correlates closely with the distribution of neuritic plaques across these same layers, with both showing greater involvement of layers III–VI than of layers I-II. Of even greater interest, normal individuals—without Alzheimer disease and without neuritic plaques—show a similar (but much less pronounced) variation in content of (resting) microglia across these cortical layers. This latter finding suggests that the normal distribution of microglia across cerebral cortical layers may influence the pattern of development of neuritic plaques in Alzheimer brain [34].

The association of activated microglia with A*β* plaques is also a function of plaque type. The A*β* plaques present in Alzheimer brain show different morphological appearances, which are thought to represent different stages of plaque progression, commencing with diffuse deposits of A*β* peptide, progressing to complex plaques with congophilic amyloid and with evidence of damage to neurons and their processes (“dystrophic neurites”), and terminating in a dense core of condensed amyloid without diffuse A*β* peptide and without adjacent dystrophic neurites [32, 35]. This schemata is widely (but not universally [7]) accepted.

Both the numbers of microglia and the degree of activation of microglia vary with plaque type, or stage [32]. Early plaques in Alzheimer brain—those which contain diffuse deposits of A*β* peptide without formation of true (fibrillar) amyloid and without evidence of neuritic injury—already contain small numbers of microglia that overexpress interleukin-1 (IL-1) [32]. These microglia are not enlarged or phagocytic [36] and thus are easily mistaken for normal “resting” microglia in the absence of specific immunohistochemical demonstration of their cytokine upregulation [26]. Of interest is the fact that similar-appearing diffuse deposits of A*β* peptide can sometimes be found, in the absence of the later plaque forms and in the absence of clinical dementia. This includes occasional otherwise normal, cognitively intact elderly individuals, and includes individuals with the disease hereditary cerebral hemorrhage with amyloidosis (Dutch) (HCHA-D), a disease that is characterized by extensive amyloid angiopathy and consequent cerebral hemorrhage. In contrast to the ubiquitous presence of activated microglia in diffuse plaques present in Alzheimer brain [32], activated microglia have not been identified in either the similar-appearing deposits in the brains of neurologically normal elderly individuals [37] or in the similar-appearing deposits in the brains of patients with HCHA-D [23]. This observation suggests that A*β* deposition alone is not sufficient for the development of Alzheimer disease. Further, this observation suggests that activated microglia, overexpressing IL-1, may be a key element in A*β* plaque progression, and hence a key element in the clinical conversion to, and progression of, clinical Alzheimer disease.

It remains unclear why the diffuse A*β* deposits in normal elderly or in HCHA-D differ in their ability to attract and activate microglia. One possibility is the lack of some codeposited, immunogenic protein, possibly originating outside the brain. In support of this idea is the observation that Alzheimer brain shows diffuse immunoreactivity for the serum-derived proteins fibrinogen and IgG, suggesting breakdown of blood-brain barrier function in Alzheimer disease [38]. This fibrinogen immunoreactivity is enhanced in A*β* deposits and is associated with microglial activation [38]. A second possibility is that there is a difference in some physical characteristic of the A*β* peptide (e.g., oligomer or fibrillar state), as may be the case for patients with HCHA-D. Finally, for those unusual elderly individuals with only diffuse A*β* deposits, there is the third possibility that there is an inherent difference in the responsiveness of microglia in these individuals.

Early, diffuse plaques progress to neuritic forms, characterized by condensation of A*β* peptide into true, fibrillar amyloid, and by the appearance of injured (dystrophic) neural processes (neurites). At this stage, there are increases both in the number of microglia associated with individual plaques, and in the degree of activation of these associated microglia [32, 36, 39]. In addition to overexpression of IL-1, these microglia become enlarged with fewer ramified processes, thus meeting classic morphological criteria (in addition to immunohistochemical criteria) for activation. With continued condensation of A*β* peptide, a “dense core” of amyloid is formed. This process appears to mask or reverse the immunogenic properties of the plaque, as the numbers of microglia associated with such plaques are less than that seen prior to the formation of the dense core [32]. Further, the microglia found in these cored plaques are phagocytic in appearance [36], although the appearance of microglia in these later plaques has also been interpreted as a degenerative or senescent change [26]. Microglia are clearly capable of phagocytosing and even removing A*β*, as trials of A*β* vaccines have suggested in both animals and humans [40].

The final, end-stage of plaque progression is a solitary dense core of amyloid, without surrounding diffuse A*β* peptide and without associated dystrophic neurites. These end-stage plaques are also devoid of microglia, suggesting that the immunogenic stimulus that attracts and activates microglia has abated [32]. This waxing and waning of numbers of activated microglia with plaque progression has its parallel in the finding that microglia in Alzheimer brain appear early in the disease course (as measured by premortem assessments using the Blessed test score) and decline late in the disease [41], at which point there is severe dementia from the accumulated neuronal loss. A similar observation is that there is a slight decrease in microglial activation in end-stage (Braak and Braak stage VI) Alzheimer brain, after showing increases over the first five Braak and Braak stages [42].

The concurrent absence of microglia and dystrophic neurites in end-stage, dense-core plaques also suggests that plaque-associated microglia may play a role in damaging surrounding neural elements and generating the dystrophic neurites of Alzheimer amyloid plaques. Interleukin-1 is known to be neurotoxic at high concentrations, as coculture of lipopolysaccharide-activated microglia with primary rat cortical neurons results in neuronal death, and this effect is attenuated in the presence of the naturally occurring IL-1 receptor antagonist IL-1ra [43]. In human brain, the association of activated, IL-1-expressing microglia with A*β* plaques correlates with histochemical evidence of progressive neuronal damage, as assessed using the TUNEL technique [44]. Cortical neurons contained within or adjacent to A*β* plaques frequently show TUNEL positivity, and the percentage of such neurons that are positive by this technique increases with plaque progression, reaching nearly 100% in end-stage dense core plaques. Further, the total number of plaque-associated neurons declines significantly in such end-stage plaques, suggesting that there is neuronal death and loss associated with plaque progression [44]. Plaques are generally much more numerous than tangles in Alzheimer brain, and neuronal loss in Alzheimer brain has long been known to exceed the incidence of neurofibrillary tangles [45]. Thus, such plaque-associated neuronal injury and loss, mediated at least in part by microglia-derived cytokine overexpression, may be a significant—and perhaps even major—source of neuronal loss in Alzheimer brain.

## 4. Microglial Associations with Neurofibrillary Tangles

Neuropathological investigations have also offered evidence that microglia may be involved in the pathogenesis and progression of neurofibrillary tangle formation. Just as there are correlations between the distribution of activated microglia and distribution of A*β* plaques in Alzheimer brain, so, too are there correlations between the distribution of microglia and the distribution of neurofibrillary tangles [46]. Further, and again as for A*β* plaques, a progressive association has been shown between activated microglia and neurofibrillary tangle stage [47]. Neurofibrillary tangles can be classified into different stages, representing a pathological progression [47, 48]. In Alzheimer brain, there is a progressive association of activated, IL-1-overexpressing microglia with neurofibrillary tangles across this spectrum of tangle progression [47]. *In vitro* work has shown that IL-1 increases neuronal expression of the tau-phosphorylating enzyme MAPK-p38 and also increases the phosphorylation (activation) of this enzyme [43]. This, together with the neuropathological observations, suggests that neuron-associated microglia may be important in driving or sustaining neuronal tangle formation in Alzheimer disease.

## 5. Microglia in Other Neurodegenerative Diseases

Chronic microglial activation as a potentially neurotoxic driving force is not likely to be restricted to Alzheimer disease. Following the seminal observations of microglial activation in Alzheimer brain, similar microglial activation has been found in other chronic neurological diseases, including amyotrophic lateral sclerosis, spinocerebellar ataxia, and Huntington's disease [49–52], and especially in diseases characterized by *α*-synuclein pathology: Parkinson's disease, dementia with Lewy bodies, and multiple system atrophy. Activated, cytokine-overexpressing microglia are found in the substantia nigra of Parkinson patients (for reviews, see references [53–55]). Activated microglia also show close associations with inclusion-bearing neurons in dementia with Lewy bodies [56] and with inclusion-containing oligodendrocytes in multiple system atrophy [57]. These findings suggest a common final pathway of neuronal injury and loss in a number of chronic neurodegenerative diseases [58].

## 6. Origins of Neuroinflammatory Processes

 Alzheimer disease, and Alzheimer-type neuropathological changes, show important associations with a number of other conditions. These include ageing, head injury, diabetes, heart disease, and epilepsy. All of these conditions have been shown to enhance or accelerate microglial activation in the brain. In normal human ageing, for instance, there is a progressive increase in the numbers of activated microglia overexpressing IL-1 [59]. This effect may serve to lower the threshold required to subsequently initiate disease [60] and may thus explain in part the strong age association of Alzheimer disease. Similarly, head injury results in activation of microglia overexpressing IL-1, together with expression of the A*β* precursor protein by neurons and neuronal processes [61]. For patients with established Alzheimer disease, and microglial activation, there is further enhancement of this microglial activation in the presence of concurrent diabetes [62] or heart disease [63]. Patients with chronic intractable temporal lobe epilepsy or with HIV infection show increased proinflammatory activation of microglia [64, 65], and both of these conditions have been associated with accelerated appearance of age-associated, Alzheimer-type “senile” changes [66, 67]. Collectively, these findings suggest that proinflammatory processes of various etiologies can contribute to a summative inflammatory state—characterized by microglial activation—that lowers the threshold and increases the risk for the subsequent development of neurodegenerative disease.

## 7. Conclusion

Microglial activation is a universal feature of Alzheimer disease, as well as a number of other chronic neurodegenerative diseases. These microglia show specific patterns of association with the neuropathological features of Alzheimer disease, which collectively suggest a pathogenic role in driving the progression of such pathology. Further, microglial activation is a prominent feature of several other conditions that have been associated with increased risk for Alzheimer disease or with accelerated appearance of Alzheimer-type neuropathological changes.
